# Developmental and tissue‐specific roles of mammalian centrosomes

**DOI:** 10.1111/febs.17212

**Published:** 2024-06-27

**Authors:** Charlotte Meyer‐Gerards, Hisham Bazzi

**Affiliations:** ^1^ Department of Cell Biology of the Skin, Medical Faculty University of Cologne Germany; ^2^ Department of Dermatology and Venereology, Medical Faculty University of Cologne Germany; ^3^ The Cologne Cluster of Excellence in Cellular Stress Responses in Aging‐associated Diseases (CECAD), Medical Faculty University of Cologne Germany; ^4^ Graduate School for Biological Sciences University of Cologne Germany; ^5^ Center for Molecular Medicine Cologne (CMMC), Medical Faculty University of Cologne Germany; ^6^ Present address: Cell & Developmental Biology University of Michigan Medical School Ann Arbor MI USA

**Keywords:** 53BP1, cell division, centriole, mitotic surveillance pathway, mouse, p53, USP28

## Abstract

Centrosomes are dominant microtubule organizing centers in animal cells with a pair of centrioles at their core. They template cilia during interphase and help organize the mitotic spindle for a more efficient cell division. Here, we review the roles of centrosomes in the early developing mouse and during organ formation. Mammalian cells respond to centrosome loss‐of‐function by activating the mitotic surveillance pathway, a timing mechanism that, when a defined mitotic duration is exceeded, leads to p53‐dependent cell death in the descendants. Mouse embryos without centrioles are highly susceptible to this pathway and undergo embryonic arrest at mid‐gestation. The complete loss of the centriolar core results in earlier and more severe phenotypes than that of other centrosomal proteins. Finally, different developing tissues possess varying thresholds and mount graded responses to the loss of centrioles that go beyond the germ layer of origin.

## Graphical abstract

Centrosomes are dominant microtubule‐organizing centers in animal cells that are required for efficient mitoses to ensure normal embryonic development. Mammalian cells respond to centrosome loss‐of‐function by activating the mitotic surveillance pathway, a timing mechanism that leads to 53BP1‐, USP28‐, and p53‐dependent cell death or arrest when a defined threshold is exceeded. Mouse embryos without centrosomes undergo embryonic arrest at mid‐gestation (E9) whereas different developing tissues mount graded responses to centrosome loss (≥ E15).

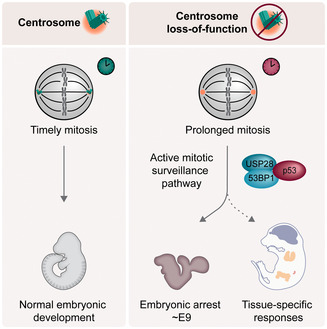

**Abbreviations**
53BP1: p53 binding protein 1
ANKRD26: Ankyrin repeat domain‐containing protein 26
BRCT: BRCA1 C‐Terminus
BSC‐1: African green monkey kidney cells
CASP2: Caspase‐2
CDK5RAP2: Cyclin‐dependent kinase 5 regulatory subunit‐associated protein 2
CDNK2A: Cyclin‐dependent kinase inhibitor 2A
CENPJ: Centromere protein J
CEP120: Centrosomal protein of 120 kDa
CEP135: Centrosomal protein of 135 kDa
CEP152: Centrosomal protein of 152 kDa
CEP192: Centrosomal protein of 192 kDa
CEP97: Centrosomal protein of 97 kDa
CETN2: Centrin‐2
CP110: Centriolar coiled‐coil protein of 110 kDa
ERK: Extracellular‐signal‐Regulated Kinase
iPSCs: induced pluripotent stem cells
MCPH: Autosomal recessive primary microcephaly
MDM2: E3 ubiquitin‐protein ligase Mdm2
MSP: Mitotic surveillance pathway
MTOCs: microtubule‐organizing centers
NPC: Neuronal progenitor cells
PCM: Pericentriolar material
PIDD1: p53‐induced death domain‐containing protein 1
PLK1: Polo‐like kinase 1
PLK4: Polo‐line kinase 4
RAIDD: RIP‐associated protein with a death domain
RPE‐1: Retinal pigment epithelial cells
SAS‐4, SASS6: Spindle assembly abnormal protein 4 or 6 homolog
shRNA: short hairpin RNA
STIL: SCL‐interrupting locus protein homolog
USP28: Ubiquitin‐specific peptidase 28
VZ: Ventricular zone

## Introduction

**Fig. 1:**
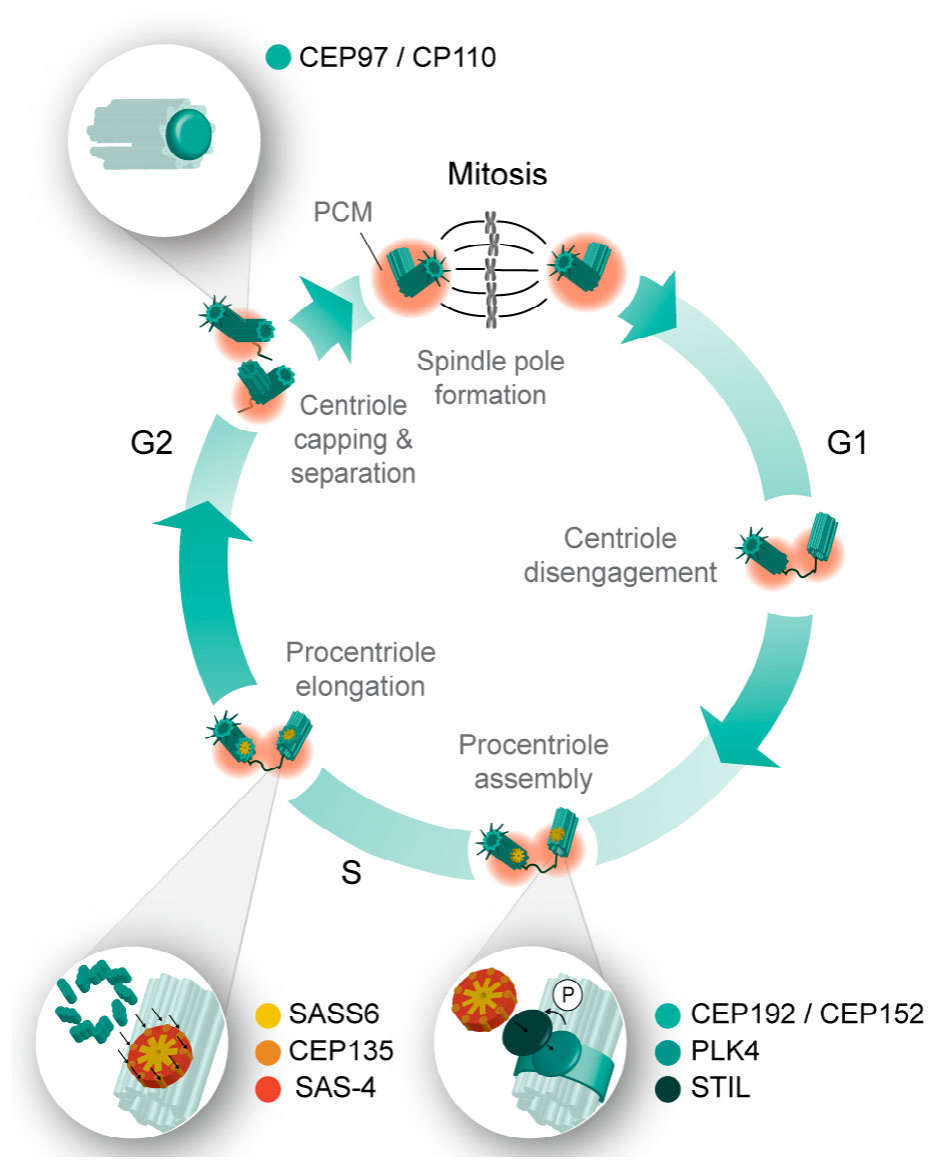
Schematic representation of the centrosome duplication cycle. To duplicate, the centrioles (cyan) disengage in late M‐G1‐phases of the cell cycle. Procentrioles start to assemble towards the end of G1‐S‐phases through the formation of the cartwheel (yellow): The pericentriolar material (PCM) proteins CEP152 and CEP192 recruit PLK4 to the proximal site of each pre‐existing centriole, where PLK4 phosphorylates STIL to recruit SASS6, CEP135 and SAS‐4 to initiate procentriole assembly. Procentrioles elongate throughout the S‐G2‐phases via the attachment of centriolar microtubules and are capped by CP110 and CEP97, when they reach a defined length. During Mitosis, the daughter centriole of the old pair is further modified to become itself a new mother centriole. Both centrosomes also recruit significantly more PCM (orange) to form the two spindle poles during mitosis.

Centrosomes are membrane‐less organelles that serve as microtubule‐organizing centers (MTOCs) in animal cells [1]. Each cell in G1 possesses a centrosome composed of a core of two orthogonal microtubule‐based centrioles, a mother and a daughter centriole, which are connected by a fibrous linker and surrounded by a proteinaceous pericentriolar material (PCM) (Fig. 1) [2]. The microtubules of mammalian centrioles are arranged in a nine‐fold symmetric cylinder of triplets, which can be divided into proximal, central, and distal regions [3, 4]. The proximal region is mainly characterized by additional A‐C linkers that connect neighboring microtubules [5, 6], and a scaffolding cartwheel, which is important for the initial assembly of centrioles but absent in fully mature centrioles of most organisms [7, 8]. The central or core region is stabilized by a helical structure termed the inner scaffold [9], whereas the distal region is decorated with subdistal appendages, which enable microtubule nucleation and anchoring [10], and distal appendages, which tether the centriole to the membrane to template cilia [4, 11, 12]. Notably, only the mature mother centriole of the pair is decorated with the appendages, while the younger daughter centriole acquires them during the next mitosis to become itself a mother centriole [4, 13].

In terms of cellular functions, the two most prominent roles of centrosomes are to help organize the mitotic spindle for an efficient cell division and to provide the essential basal body centriolar template for cilia during interphase and in differentiated cells. Moreover, centrosomes organize the microtubule network that is used as the cellular roadway and helps establish polarity and directionality [2, 14, 15]. In addition, centrosomes act as signaling hubs in a cell with a plethora of associated house‐keeping biological processes including vesicular transport and proteasomal degradation [16, 17].

Similar to DNA replication, the duplication and segregation of centrosomes and centrioles is tightly controlled during the cell cycle to ensure that each daughter cell inherits one pair of centrioles after mitosis (Fig. 1) [18, 19, 20, 21]. To duplicate, the centrioles first disengage during the M‐G1‐phases of the cell cycle and the daughter centriole acquires PCM in a process termed “centriole‐to‐centrosome conversion.” During S‐phase, the PCM proteins Centrosomal protein of 152 kDa (CEP152) and Centrosomal protein of 192 kDa (CEP192) recruit Polo‐like kinase 4 (PLK4) to the proximal side of each pre‐existing centriole, marking the site of procentriole assembly [22, 23]. Next, PLK4 phosphorylates SCL‐interrupting locus protein homolog (STIL) and initiates a cascade of events, starting with the recruitment of the cartwheel‐forming protein Spindle assembly abnormal protein 6 homolog (SASS6). This is followed by Centrosomal protein of 135 kDa (CEP135), and then SAS‐4 (CENPJ, or CPAP in humans), which forms a complex with γ‐tubulin to initiate procentriole assembly [24, 25, 26, 27]. Each procentriole then elongates throughout the G2‐phase and is capped by Centriolar coiled‐coil protein of 110 kDa (CP110) and Centrosomal protein of 97 kDa (CEP97), when it reaches a defined length [28]. During mitosis, the daughter centriole of the old pair is further modified to become itself a new mother centriole [29]. Finally, the two mother/daughter centrioles separate and recruit more PCM to form the two spindle pole centrosomes during mitosis [29].

Dysregulation of centrosome duplication, ranging from partial centrosome loss‐of‐function to amplification, is evident in several human diseases including developmental primordial dwarfism and microcephaly (loss‐of‐function) and cancer (amplification) [30, 31]. Based on animal studies, the complete loss of centriole and centrosome function is likely not to be compatible with life [32]. Cells have established mechanisms to sense both scenarios of centrosome loss and amplification, mostly leading to cell cycle arrest and/or cell death. These mechanisms rely on two genetically and biochemically distinct signaling pathways, that are discussed in more detail below. In addition, accumulating evidence suggests that different tissues have differential requirements for centrosomes, resulting in variable susceptibilities to centrosome loss‐of‐function. In this review, we will highlight the recent advances in our understanding of the roles of mammalian centrosomes during development and discuss their tissue‐specific functions with an emphasis on the developing mouse, where most of the studies have been conducted.

### Centrosome amplification

**Fig. 2:**
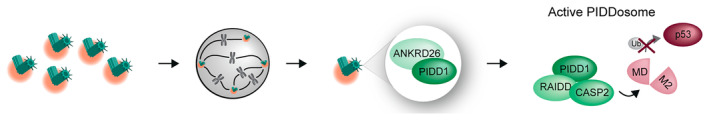
Schematic representation of the pathway activated upon centrosome amplification. Supernumerary centrosomes, each consisting of a pair of centrioles (cyan) with pericentriolar material (PCM) (orange), resulting from deregulation of centriole duplication, lead to multipolar spindle formation and increased probability of chromosome segregation errors and aneuploidy. Supernumerary centrioles are sensed by activating the multiprotein complex “PIDDosome.” First, a PIDD1 precursor is recruited by ANKRD26 to the distal appendages of the mother centriole, where it undergoes auto‐proteolysis. This primed PIDD1 is then released into the cytoplasm and activates the PIDDosome (green, composed of PIDD1, RAIDD, and CASP2). In turn, the PIDDosome cleaves and inactivates the p53‐E3‐ubiqutin ligase MDM2 (light pink), resulting in p53 (burgundy) de‐ubiquitination and stabilization.

Centriole and centrosome amplification, which is tightly associated with cancer, results from deregulation of centriole duplication and leads to multipolar spindle formation and increased probability of chromosome segregation errors and aneuploidy (Fig. 2) [19, 31, 33, 34, 35, 36]. For example, the overexpression of PLK4 can be sufficient to induce centriole amplification and cancer in mice [37, 38]. Moreover, transient overexpression of PLK4 was shown to accelerate tumorigenesis in the p53‐deficient skin epidermis [39]. In addition, PLK4 has been identified as an oncogene, which is overexpressed in various types of human cancers, and functional studies revealed that knockdown of PLK4 in colorectal cancer cells results in a significant decrease in cell viability and proliferation [40]. However, in the mouse brain, centrosome amplification causes microcephaly due to chromosome mis‐segregation in aneuploid cells that undergo apoptosis, which is only partially p53‐dependent [41]. Collectively, these results highlight that centrosome amplification together with chromosome mis‐segregation can cause tissue degeneration in developing tissues or hyperproliferation and cancer depending on the cellular context.

In non‐transformed cells, supernumerary centrioles are sensed by activating the multiprotein complex “PIDDosome” (Fig. 2) [42]. The active PIDDosome is composed of p53‐induced death domain‐containing protein 1 (PIDD1), RIP‐associated protein with a death domain (RAIDD) and Caspase‐2 (CASP2), which proteolytically inactivates E3 ubiquitin‐protein ligase Mdm2 (MDM2), resulting in p53 stabilization and subsequently cell cycle arrest or cell death. A model for how cells sense supernumerary centrioles via the PIDDosome has only recently been elucidated (Fig. 2): A PIDD1 precursor is recruited by Ankyrin repeat domain‐containing protein 26 (ANKRD26) to the distal appendages of the mother centriole, where it undergoes auto‐proteolysis and clustering with other mature mother centrioles. This primed PIDD1 is then released into the cytoplasm and the PIDDosome is activated [43, 44]. In this review, we will not focus on centrosome amplification, which has recently been reviewed in detail [31], but rather focus on centrosome loss‐of‐function, which activates a distinct p53‐dependent cell cycle mechanism.

### Centrosome loss‐of‐function

In humans, mutations in genes encoding centrosomal proteins are associated with developmental disorders characterized by primordial dwarfism and microcephaly [30, 45, 46]. How does the loss of centrosome function lead to these human manifestations? We will start with a historical journey on the major functions of centrosomes and their cell type‐specific roles, which have been extensively studied in the past few decades. In mammalian cell lines, namely cultured African green monkey kidney cells (BSC‐1), centrosome removal by microsurgery led to an arrest in G1 [47, 48]. Moreover, depletion of centrosomal proteins using siRNA in cultured non‐transformed human retinal pigment epithelial (RPE‐1) cells caused a p38‐ and p53‐dependent cell cycle arrest in G1, presumably from within G1 [49]. Further experimental evidence refuted a G1‐S transition sensing mechanism upon the loss of centrosomes and suggested that the cells experience functional stress and a p38‐dependent G1‐arrest [50]. However, the exact phase of the cell cycle where centrosome loss was sensed and led to the cell cycle arrest was not precisely determined due to technical limitations in following the cells without centrosomes through multiple cell cycles.

**Fig. 3:**
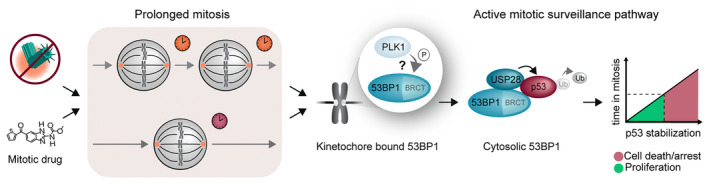
Schematic representation of the mitotic surveillance pathway activated after centrosome loss and/or prolonged mitosis. Centrosome loss or the use of mitotic drugs prolongs mitosis and activates the mitotic surveillance pathway (MSP). A single long mitotic duration (bottom in the box, burgundy clock) or successive sub‐threshold mitotic durations (top, orange clocks) induce MSP activation through a PLK1‐facilitated interaction of 53BP1, USP28, and p53, possibly via phosphorylation of 53BP1 and/or other substrates, culminating in the de‐ubiquitination and stabilization of p53. The level of p53 stabilization correlates with the time spent in mitosis and, when a defined threshold is exceeded, leads to cell death or cell cycle arrest after exit from mitosis.

In a mouse knockout study, genetic removal of *Sas‐4* (*Cenpj* or *Sas4*) caused centriole loss and embryonic lethality at mid‐gestation (see below) [51]. Time‐lapse imaging in mouse embryos showed that the loss of centrosome function in *Sas‐4* mutants was associated with prolonged mitosis, and led to the activation of a p53‐dependent apoptosis pathway (Fig. 3). Importantly, the stabilization of p53 upon centriole loss was largely independent of DNA damage repair pathways or chromosome segregation errors, major culprits in p53‐mediated stress responses [51].

In a previous and elegant study, treatment of RPE‐1 cells *in vitro* with mitotic drugs extended mitotic duration and induced p38‐ and p53‐dependent G1 cell cycle arrest (Fig. 3) [52]. Interestingly, the G1 arrest was also rescued by transiently inhibiting p38; however, the granddaughter cells still arrested later even though they were born from mitoses of normal duration [52]. To assess whether a brief mitotic arrest can cause p53 upregulation and cell death in mouse embryos similar to centriole loss, wild‐type mouse embryos were treated with mitotic drugs such as nocodazole, which led to p53 stabilization and induced cell death after drug washout [51]. Therefore, these findings linked the centrosome loss‐dependent cell cycle exit to prolonged mitotic duration, suggesting that the p53‐dependent apoptosis pathway senses the duration of mitosis and acts as a “mitotic timer” (Fig. 3). Two subsequent reports *in vitro* established that the loss of centrioles using PLK4 inhibition or degradation in RPE‐1 cells caused p53‐dependent cell cycle arrest [53, 54]. Although these studies mostly ruled out the involvement of the DNA damage or aneuploidy in the response to centriole loss, the definitive link to prolonged mitotic duration was still controversial or missing.

Since the connection between centrosome loss‐of‐function and p53‐dependent downstream responses, p53 binding protein 1 (53BP1) and ubiquitin‐specific peptidase 28 (USP28) have been identified as essential in the signaling cascade upstream of p53 (Fig. 3). Using CRISPR/Cas9 short hairpin RNA (shRNA) library screens in RPE‐1 cells that were depleted of centrioles by targeting PLK4, three groups independently identified upstream players of the pathway including 53BP1 and USP28 [55, 56, 57]. Notably, although both proteins are known to play roles in the DNA damage repair [58, 59, 60, 61], their roles in this pathway, similar to that of p53 in the earlier reports, were shown to be independent of their involvement in the DNA damage response [62]. Furthermore, both p53 and USP28 have been shown to interact with the two BRCA1 C‐Terminal (BRCT) domains of 53BP1 (Fig. 3) [58, 63, 64], which are essential for the upregulation of p53 in response to centrosome loss and prolonged mitosis [55, 65]. USP28 also interacts with the Tandem‐Tudor domain of 53BP1 in interphase as well as upon MSP activation [66, 67]. Thus, 53BP1 seems to act as a scaffold, which brings USP28 and p53 into close proximity in order to allow USP28‐mediated de‐ubiquitination and stabilization of p53 (Fig. 3) [55, 67, 68]. The pathway was termed “the mitotic surveillance pathway” (MSP) and, more recently, “the mitotic stopwatch” [62, 67] (Fig. 3). Recent work has shown that 53BP1 and USP28 are also essential for the activation of the MSP *in vivo* in the developing mouse embryo and brain [69, 70].

The mitotic timer model is further supported by additional lines of evidence. First, another hit in the CRISPR screens was *Trim37*, an E3‐ubiquiting ligase whose mutation rescues the proliferative defect in RPE‐1 cells without centrioles by creating ectopic MTOCs and accelerating mitosis [57]. In this context, the loss of *Trim37*, and unlike that of *53bp1*, *Usp28* or *p53*, fails to prevent cell cycle arrest when the cells are forced to arrest in mitosis using pharmacological agents. Second, in RPE1 cells, the duration spent in mitosis correlates with a progressive increase of the p53‐downstream cell cycle inhibitor p21 in G1, and only the cells reaching a defined threshold arrest the cell cycle [57, 67]. Finally, RPE‐1 cells experiencing successive sub‐threshold mitotic durations arrest after multiple cell cycles [67].

Remarkably, cultured primary mouse embryonic stem cells are able to grow without centrioles under pluripotent conditions; however, they reveal an increasing growth defect under partially differentiated conditions, suggesting that the threshold for activating the MSP can differ depending on the cellular context [70]. While the loss of centrosome function induces p53‐dependent G1 arrest, cell lines with mutations in p53 or Cyclin‐dependent kinase inhibitor 2A (CDNK2A) continue to proliferate [54]. Interestingly, two neuroblastoma‐derived cell lines exhibit cell death instead of arrest upon centrosome loss, which is similar to the findings in mouse embryos *in vivo* [51, 67]. Collectively, centrosome loss and extended mitotic duration trigger the activation of the MSP, which monitors the duration of mitosis; however, both the degree or threshold of the mitotic duration as well as the subsequent cell fates of G1 arrest or apoptosis differ between cell types (Fig. 3).

Recently, Polo‐like Kinase 1 (PLK1), which was reported to phosphorylate 53BP1 in high‐throughput studies [71, 72], has been identified to act upstream of 53BP1 by displacing 53BP1 from the kinetochores during mitosis and enabling the subsequent interaction of 53BP1 with p53 and USP28 (Fig. 3) [67, 73]. In this respect, the expression of a 53BP1 transgene with three putative PLK1‐dependent phosphorylation sites (T1756, S1758, and S1759), mutated to Alanine, disrupted the interaction between 53BP1 and p53 during prolonged mitosis and reduced p53 stabilization in human RPE‐1 cells [67]. Moreover, the E‐ubiquitin ligase MDM2, which regulates p53 levels, has been shown to be degraded during prolonged mitosis, correlating with p53 stabilization and cell cycle arrest [74]. However, it is unlikely that this is the sole sensor for mitotic duration because MG132 activates the MSP while inhibiting the proteasome [55], and preventing MDM2 degradation, which argues against MDM2 stability being the mitotic timer for MSP activation. To date, the exact mechanism by which the cells sense centrosome loss and/or prolonged mitotic duration and the potential involvement of additional proteins mediating this signaling pathway remain unknown.

## Centrosomes during early mammalian development

Most mammalian species rely on the centrioles and centrosomes during the early cell divisions with an exception in rodents, which perform the first cleavage divisions in the absence of centrioles [75, 76, 77]. The zygotes of most other studied mammalian species, including humans, inherit their centrioles from the sperm [78, 79]. The sperm has a typical and an atypical centriole, both of which can participate in spindle pole formation in the zygote [80, 81, 82]. The centrioles of the mouse sperm are only transiently detected during mid‐spermatogenesis and undergo remodeling until they disappear in the mature spermatozoa [76, 77, 81, 83]. Recent advances in ultrastructural imaging techniques performed with bovine embryos showed that the typical centriole received from the sperm disappears after the first division, while new centrioles formed *de novo* after two divisions [84]. Hence, the mechanism of *de novo* centriole formation seems to go beyond rodents and the timing of centriole biogenesis varies.

### Centrosomes during preimplantation

Around E3 in the mouse, the centrioles start to form *de novo* [85, 86, 87], but the newly formed centrioles do not seem to participate in microtubule nucleation in interphase cells [88]. Three decades ago, it was notably observed that the microtubule‐nucleating protein γ‐tubulin focally accumulates in interphase cells at the morula stage in GFP::CETN2 transgenic mice [87], where Centrin‐2 (CETN2) marks the centriolar inner walls, suggesting that centrioles form within aggregates of existing PCM. Given that centrioles in the mouse do not form until E3, the early cell fate decisions and lineage specifications in the embryo do not depend on centrioles but rather on acentrosomal MTOCs and other mechanisms, including the position of the cells within the embryo and intercellular communication [85, 89]. Recently, it has been shown that the heterogeneities in cell polarity trigger the assembly of a mono‐astral spindle which in turn induces spatially asymmetric division patterns [90]. The possible advantages that led to the development of mouse embryos without sperm‐derived centrioles may be attributed to peculiarities of mouse development. For example, Gueth‐Hallonet *et al*. suggested that the random position of the spindle during the 4‐cell cleavage and the irregular cleavage planes or detectable axes of polarity before the 8‐cell stage, may be linked to the lack of centrioles [87, 91]. Remarkably, cells of the mouse inner cell mass retain the ability to differentiate into trophectoderm [92], and individual blastomeres of 16‐ or 32‐cell embryos are able to develop into viable mice [93], revealing a high degree of plasticity and flexibility during early mouse development.

It is interesting to note that the requirement for centrioles during early development varies among different species as well as developmental stages. For example, *Sas‐4* depletion and centriole loss leads to developmental arrest of *C. elegans* at the two‐cell stage [94]. In *D. melanogaster*, zygotic *Sas‐4*‐mutant flies, with maternal *Sas‐4* mRNA during the early divisions, can develop without centrioles until adulthood, when they die due to the lack of cilia in their sensory neurons [95]. However, when the maternal pool of *Sas‐4* is depleted, the embryos arrest development at the syncytial division stage [96].

### Centrosomes post‐implantation

**Fig. 4:**
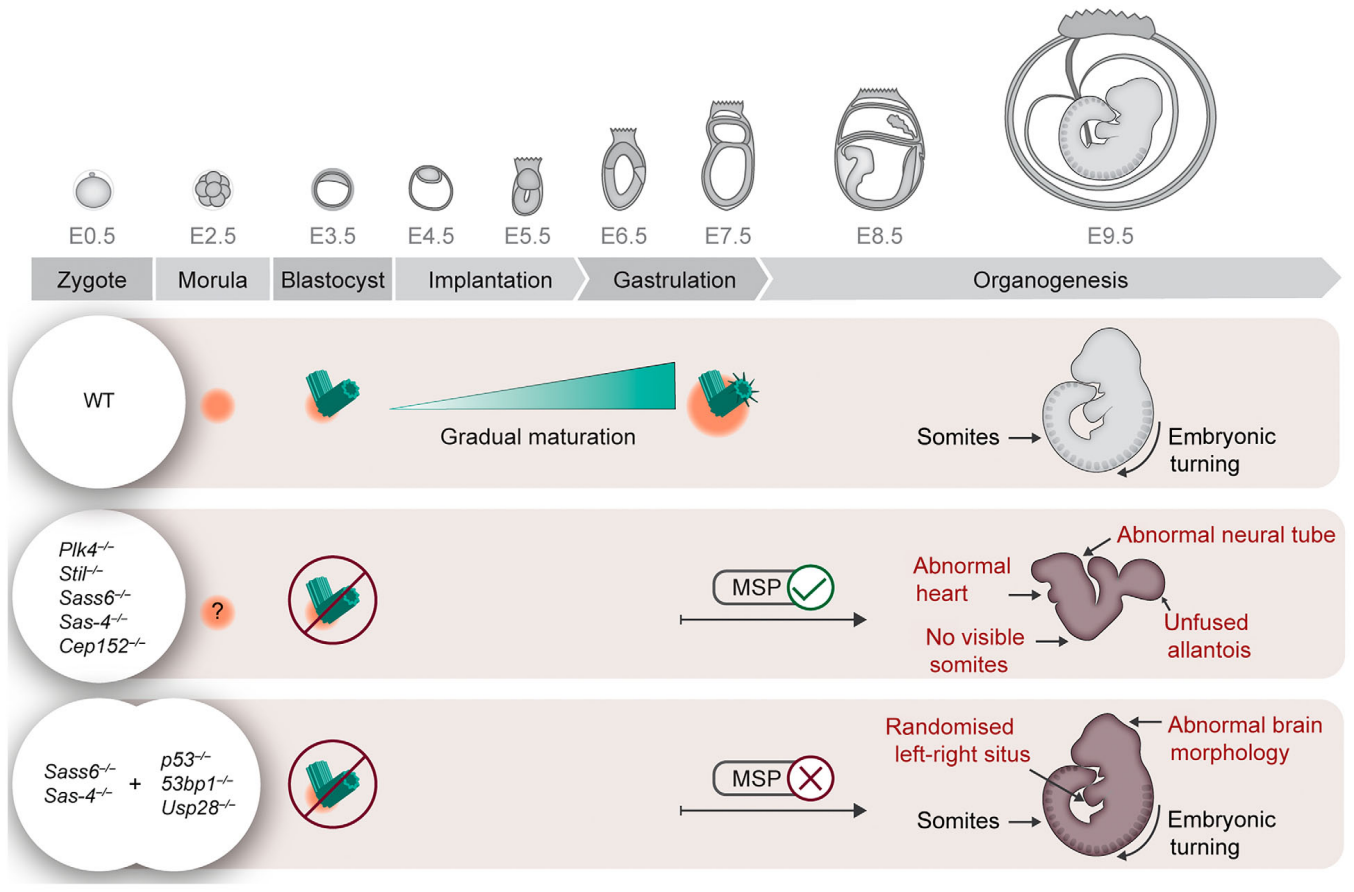
Schematic representation of centriole maturation and the consequences of centrosome loss during mouse embryonic development. Centrioles (cyan) first form by *de novo* biogenesis in the mouse blastocyst around embryonic day (E) 3 within aggregates of pericentriolar material (PCM) (orange). They gradually mature, acquire appendages (spokes), and recruit more PCM. Mutations in genes that are essential for centriole and centrosome formation (*Plk4*, *Stil*, *Sass6*, *Sas‐4* or *Cep152*) lead to the loss of centrioles, the activation of the mitotic surveillance pathway (MSP) around E7 and ultimately embryonic arrest around mid‐gestation (~ E9). The mutant embryos still manage to develop a heart and neural tube but have an unfused allantois. They also fail to undergo embryonic turning or show visible somites, which are typical landmarks in wild‐type (WT) embryos. The genetic removal of any of the players in the MSP (*p53*, *53bp1*, or *Usp28*) in addition to *Sas‐4* or *Sass6* rescues the cell death and allows the double mutant embryos to develop further, when they resemble cilia mutants with an abnormal brain morphology and randomized left–right heart looping or situs. Abnormal developmental features are displayed in a red font. The embryo development timeline was adapted and modified with permission from [162]. Copyright © 2002 The Protein Society.

As the cells of the developing mouse embryo differentiate into more defined lineages, centrioles become increasingly important and eventually essential for cellular and organismal survival [51]. In this context, the newly formed centrioles are immature, lack appendage protein markers and recruit less PCM in interphase [70]. Importantly, the newly formed centrioles do not seem to be competent to function as MTOCs during mitosis because embryos lacking centrioles have a similar mitotic fraction to wild‐types [70]. The maturing centrioles gradually acquire appendage proteins until the onset of gastrulation around E6.5, when they recruit more PCM in interphase and contribute to centrosome maturation during mitosis (Fig. 4) [70]. The course of centriole maturation also correlates with the appearance of cilia in cells of the epiblast and the formation of the three germ layers ~ E6 [97]. How centrioles form *de novo* in the developing mouse embryo and how they mature over time and become competent MTOCs, remain open questions.

The importance of centrioles and centrosomes during mammalian development is clearly evident in the severe phenotypes observed upon their loss (Fig. 4 and Table 1). Several groups have reported cell death and mouse embryonic arrest resulting from disrupted centriole biogenesis and duplication via inactivation of *Plk4*, *Stil*, *Rttn*, *Cep152*, *Sas‐4*, *or Sass‐6*. Except for *Sas‐4*, *Sass‐6*, and *Cep152*, it was not known at the time that the other genes were essential for centriole formation or duplication [51, 98, 99, 100, 101, 102]. Of note, the loss of centrosomal components, which are non‐essential for centriole biogenesis *per se*, including but not limited to, distal lumen and distal appendage proteins, as well as the loss of PCM components, cause milder phenotypes resembling the loss or malfunction of cilia (Table 1) [103, 104, 105, 106, 107, 108, 109, 110, 111, 112].

**Table 1:** The mouse phenotypes resulting from the loss of different centrosomal genes. The most severe phenotypes (top) including cell death and early mouse embryonic arrest are observed upon the loss of genes that are essential for centriole biogenesis and duplication. The loss of centrosomal components, which are non‐essential for centriole biogenesis *per se*, cause milder phenotypes encompassing and resembling the loss of cilia. For details on the localization of centrosomal and pericentriolar material (PCM) proteins (highlighted in burgundy) see [29]. Phenotypes are described as annotated in the Mouse Genome Database [163].

Gene	Synonyms	Protein localization	Mouse phenotype (in order of severity)
*Plk4*	*Sak*, *Stk18*	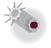 Procentriole	Embryonic lethal after the beginning of gastrulation at E7.5 with increased number of mitotic and apoptotic cells [98]
*Cenpj*	*Sas4*, *Sas‐4*, *Cpap*	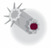 Procentriole	Embryonic lethal at mid‐gestation with elevated levels of p53 and increased apoptotic cell death [51]
*Sass6*	*Sas6*, *Sas‐6*	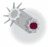 Procentriole	Embryonic lethal at mid‐gestation with elevated levels of p53 and increased apoptotic cell death [100]
*Cep152*	*Kiaa0912*	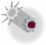 Procentriole	Embryonic lethal at mid‐gestation with elevated levels of p53 and increased apoptotic cell death [51]
*Stil*	*Sil*	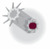 Procentriole	Embryonic lethal around mid‐gestation with axial midline defects, a block in midline Sonic hedgehog (Shh) signaling and randomized cardiac looping [99]
*Rttn*	*Ana3*	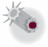 Procentriole	Embryonic lethal at mid‐gestation associated with randomized heart looping, delayed neural tube closure and failure to undergo axial rotation [101, 102]
*Cep164*	*Kiaa1052*	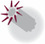 Appendages	Embryonic lethal after E10.5, characterized by holoprosencephaly, cardiac looping defects, an edematous pericardial sac and a truncated posterior trunk [103]
*Pcnt*	*Pcnt2, Kendrin*	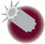 PCM	Embryonic lethality between E15.5 and E17.5. with growth retardation, microcephaly, vascular anomalies and heart defects [104]
*Cp110*	*Ccp110*, *Cep110*, *Kiaa0419*	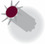 Distal cap	Lethal shortly after birth due to organogenesis defects as in ciliopathies [105]
*Cdk5rap2*	*Kiaa1633*	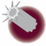 PCM	Lethal neonatally with microcephaly and defects in multiple organs including thymus and testis [106]
*Cep135*	*Cep4*, *Kiaa0635*	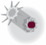 Procentriole	Lethal neonatally with microcephaly and defects in multiple organs including intestine, lungs and retina [112]
*Cby*	*Cby1*, *Pgea1*	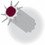 Distal end	*Mostly lethal before or around weaning with rhinitis and sinusitis* [107]
*Cep63*	*D9Mgc48e*	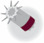 Proximal end	Viable with growth defects and microcephaly as well as male infertility [111]
*Cetn1*	*‐*	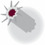 Distal lumen	Male infertility [108]
*Cetn2*	*Calt*	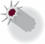 Distal lumen	Partial prenatal lethality in males and p‐ups are born with normal size but show some ciliopathy defects [109]
*Akap9*	*Akap450, Kiaa0803*	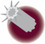 PCM	Male infertility [110]

Strikingly, each of the reported mouse mutations that cause a major loss of centrioles lead to developmental arrest around mid‐gestation (Fig. 4) [32]. Due to the essential requirement of centrioles in templating cilia and the established roles of primary cilia for the conduction of Hedgehog signaling in the developing mouse [113], the phenotypes are also associated with cilia and Hedgehog signaling defects [51, 98, 99, 101, 102]. Whereas the disruption of Hedgehog signaling is attributed to the lack of primary cilia, the cell death is specifically linked to prolonged mitosis and the activation of the MSP [51].

Remarkably, the ablation of *p53*, *53bp1*, *or Usp28* in addition to *Sas‐4* or *Sass6* rescues the cell death and allows the double mutant embryos to develop 1 day further, when they resemble cilia mutants (Fig. 4) [51, 70, 100]. Nonetheless, the embryos without centrioles manage to form a heart and neural tube but fail to undergo embryonic turning, while the double mutants proceed through embryonic turning, develop visible somites and show a characteristic abnormal brain morphology associated with impaired Hedgehog signaling [51, 70]. In summary, the primary function of centrioles and centrosomes post‐implantation in the mouse is likely to facilitate efficient and timely mitoses around the beginning of gastrulation, when proliferation rates increase, with average cell cycle times of 4–8 h [114], compared to 12–24 h during the first divisions [115]. It remains to be elucidated whether the duration of mitosis during the rapid cell division cycles determines the threshold requirement for centrosome function in other species.

## Centrosomes in developing tissues

Centrosomes function as the dominant MTOCs in proliferating animal cells, while the dependence on non‐centrosomal MTOCs is a hallmark of differentiation [116]. For example, differentiated epithelial cells, cardiomyocytes, skeletal myotubes, and neurons exhibit dominant MTOCs at non‐centrosomal sites such as cell junctions, nuclear envelope, golgi, and existing microtubules [116]. Other cell types such as keratinocytes continue to generate microtubules at the centrosome that are not bundled there [116, 117, 118]. The tissue‐specific roles of mouse centrosomes have been mainly investigated during development and organogenesis. The functions of cilia, which do not form upon the loss of centrioles, will not be covered in this review because they have recently been elegantly and exhaustively reviewed elsewhere [119, 120, 121].

**Fig. 5:**
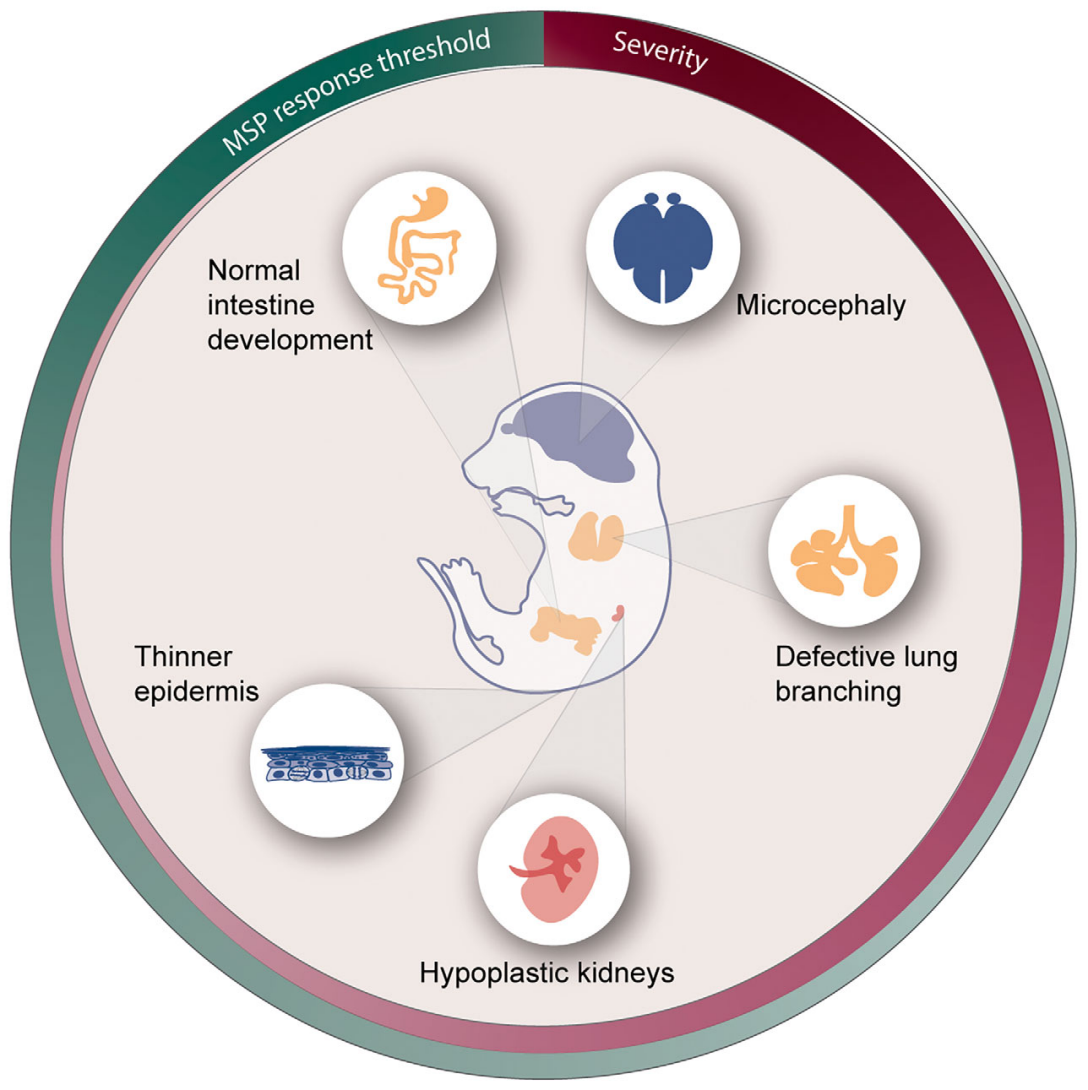
Schematic representation of the spectrum of severity of developing tissue‐specific phenotypes due to the loss of centrioles and the inverse correlation with the MSP activation threshold. The main phenotypes in the developing brain (microcephaly), lung (defective branching), kidney (hypoplasia), skin epidermis (thin), and intestine (normal) are displayed in the order of severity (burgundy circle gradient), where the brain is the most severely affected tissue. The threshold of the mitotic surveillance pathway (MSP) response (dark green circle gradient) may inversely correlate with the severity of the phenotype in the different tissues, where the brain has the lowest and the intestine the highest threshold of MSP activation. The color of the tissue represents the germ layer of origin: ectoderm (blue), mesoderm (light red), and endoderm (dark yellow). Note that the severity of the phenotype does not correlate with the germ layer.

To date, the roles of centrioles have been examined in tissues derived from all three germ layers and have mostly been studied in embryos of conditional mutant mice lacking centrioles in one specific tissue or in tissues derived from the same germ layer [69, 122, 123, 124, 125, 126]. The most prominent consequence of centriole and centrosome loss‐of‐function and ensuing prolonged mitoses is the activation of the MSP leading to cell death and/or cell differentiation aberrations. We propose that the threshold of MSP activation in different tissues and cell types inversely correlates with the severity of the phenotype (Fig. 5, see below). Interestingly, while the loss of centrioles affects the developing epiblast more than the endoderm ~ E7‐E8 [51], the response is not likely to be attributed to the germ layer of origin because tissues derived from the same germ layer display a differential susceptibility to centriole loss. This spectrum of MSP threshold versus phenotype severity in developing tissues will be reviewed below in the approximate order of how severe the tissue is affected, beginning with the most severe phenotypes likely associated with the lowest MSP thresholds (Fig. 5).

A few reports in the mouse examined centrosome functions that are independent of p53. For example, genetic mutations in the PCM protein CDK5 regulatory subunit‐associated protein 2 (CDK5RAP2) causes macrocytic anemia in mice, where the erythroid progenitors from fetal liver revealed an impaired enucleation phenotype and generated fewer and larger reticulocytes as well as highly aberrant spindle morphologies [125]. In this case, the depletion of p53 did not rescue the impaired enucleation phenotype in CDK5RAP2‐deficient erythroid progenitors [125]. In immune cells, centrosomal polarization and movement towards the immunological synapse of both cytotoxic T lymphocytes and natural killer cells has been associated with the delivery of secretory granules to the target site [127, 128]. Here, centrioles controlled the capacity, but not the specificity, of cytotoxic T cell killing in cells lacking both SAS‐4 and p53 [129].

### Centrosomes in the developing brain

A considerable body of research on the roles of centrosomes has focused on the developing brain because human mutations in genes encoding centrosomal proteins frequently manifest in autosomal recessive primary microcephaly (MCPH), a prenatal neurodevelopmental disorder that is characterized by a small brain size and a thinner cerebral cortex [30, 45, 46, 130, 131, 132]. Even though centrosomal genes are ubiquitously expressed, the brain appears to be more vulnerable than other tissues and organs to the impairment of centrosome functions [30, 133]. Thus, the brains without centrioles occupy the most severe part of the phenotypic spectrum, together with the presumed lowest threshold of MSP activation (Fig. 5). To understand the impact of mutations in centrosomal genes on MCPH, it is especially important to study the roles of centrosomes during brain development, because the neurons that contribute to cortical architecture and function are produced prenatally by a pool of neuronal progenitor cells (NPC), which are significantly depleted in microcephaly patients [45, 131, 133, 134]. NPCs undergo asymmetric divisions in the ventricular zone (VZ) of the developing neocortex to maintain the pool of NPCs while giving rise to neurons or intermediate progenitors that migrate to the subventricular zone where they will divide again [134, 135]. Of note, the resulting neuronal population is post‐mitotic and the centrosome MT nucleation capacity is gradually diminished as microtubule organization and vesicular transport start to rely on acentrosomal MTOCs [136, 137].

Insights into the roles of centrosomes during neurogenesis were first obtained from studies on the brain of the fly *D. melanogaster*. Similar to mammalian brain development, a population of neuronal stem cells, called neuroblasts, divides asymmetrically to give rise to a neuroblast and a ganglion mother cell, the latter of which divides again to produce neurons and glial cells [138]. Time‐lapse imaging revealed that the mother centrosome is inherited by the differentiating daughter cell and that the stemness properties of neuroblasts are not linked to mother centrosome inheritance [139]. While the loss of centrioles impairs spindle orientation and asymmetric divisions causing mild proliferation defects, the brain still develops relatively normally and without appreciable aneuploidy, suggesting that centrosomes are not essential during neurogenesis in the fly brain [95].

To investigate how centrosomes impact NPC cell fate in mice, Wang *et al*. [140] used an *in utero* photo‐conversion approach to distinguish between centrosomes with differently aged mother centrioles. Their data showed that the cell which inherited the older mother centriole gave rise to NPCs while the younger mother centriole was preferentially inherited by the differentiating cell [140]. Cortical neurogenesis has also functionally been investigated upon the progressive loss of centrioles in NPCs in *Sas‐4* conditional mutant mice, which displayed a prominent microcephaly phenotype by E15 (Fig. 5) [69, 141]. The loss of centrioles in NPCs was associated with NPC detachment from the VZ suggesting that centrosomes function in anchoring NPCs in the VZ [141]. Furthermore, centriole loss resulted in an apparent prolonged mitotic duration and the activation of the 53BP1‐USP28‐p53‐dependent MSP [69, 141]. In support of these findings, acutely prolonging mitosis using mitotic drugs *in vivo* before harvesting brains for fate analysis, resulted in increased apoptosis and enrichment of transcripts related to the p53 pathway during cortical development [142]. Remarkably, both apoptosis and microcephaly were rescued by the removal of *53bp1*, *Usp28* or *p53* in *Sas‐4* conditional mutants [69, 141]. In addition, NPCs in *Sas‐4 p53* double mutants retained their proliferative capacity and randomized their cleavage planes relative to the basement membrane without grossly affecting their cell fate or the resulting cortical layers [141].

Subsequent work showed that the anchorage of the centriole to the apical membrane of NPCs via its distal appendages is associated with their mechanical properties and cell fate [143, 144]. In addition, centriole maturation is a prerequisite to cilia assembly during interphase and the cell which inherits the mature mother centriole as well as the centrosome‐associated primary cilium membrane may participate earlier in cilia‐dependent signaling that further influences cell growth and cell fate decisions [145, 146, 147]. It was also suggested that additional, yet still elusive, mechanisms must exist that impact NPC cell fate decisions [30, 132, 148]. Recently, it has been proposed that centrosomes also function as an F‐actin organizer in early developing neurons, suggesting that F‐actin delivery preferentially to growth cones of the shorter neurites, the future dendrites, is involved in establishing cell polarity [149].

Another set of experiments also suggested that impaired centrosome function leads to the premature differentiation of NPCs during neurogenesis leading to less symmetric divisions and ultimately a deficit in the number of neurons, similar to that observed in microcephaly patients [30, 69, 141, 150, 151]. For example, heterotopias, ectopic layers due to premature differentiation, were observed in certain cortical areas in *Sas‐4* mutant brains [141]. However, these heterotopias might occur due to the loss of apical and basal processes connecting the NPCs with the ventricular surface and outer pial wall, respectively, which are used as roadways for neuronal migration. In addition, a mildly increased mitotic duration in NPCs induced premature neurogenic divisions [69, 142, 152, 153].

It is noteworthy that the prevalent models have been established and validated largely in genetically modified mice. Because rodents have a smooth neocortex while the human brain is characterized by a high degree of cortical folding, as well as additional progenitor cell populations, new approaches, including additional model organisms such as ferrets (*Mustela putorius furo*) and human *in vitro* brain organoids, have recently been developed [46, 154, 155]. In contrast to mice, the study of ferrets revealed a role for *Aspm*, the most common recessive microcephaly gene in humans, in regulating cortical expansion [155]. To study the role of centrosomes in human brain organoids, skin fibroblasts from a patient with severe microcephaly, who had a mutation in *CDK5RAP2*, were reprogramed into induced pluripotent stem cells (iPSCs) and propagated to form cerebral organoids in 3D‐culture [154]. These organoids recapitulated the microcephaly phenotypes compared to controls. Analyses of these organoids suggested premature differentiation because the pool of progenitor cells was depleted and the number of differentiated neurons was increased [154]. Similar phenotypes were also observed in organoids that lacked the centrosomal protein WDR62, where smaller organoids showed a decrease in NPC proliferation and premature differentiation [156]. It is noteworthy to mention that the process of organoid formation might itself involve similar developmental paths of NPCs cell death, detachment, and the likely loss of basal and apical processes, similar to developing mouse brains.

### Centrioles in the developing lung

The roles of centrosomes in the developing lung have been studied in Shh‐expressing endoderm cells depleted of centrioles through the knockout of *Sas‐4* [122]. The loss of centrioles stabilized p53 and p21 in all lung epithelial cells, but centrioles were critical for the survival of the proximal lung bronchiolar epithelial cells and branching morphogenesis (Fig. 5). These phenotypes were rescued by additionally removing p53 [122]. Moreover, the tissue and cell type‐specific sensitivity to p53 signaling in the developing lung inversely correlated with the expression and activation of Extracellular‐signal‐Regulated Kinase (ERK), where ERK activity was high in the protected distal lung cells but not in the vulnerable proximal cells. Using lung epithelial organoids revealed that the inhibition of ERK dramatically increased apoptosis in acentriolar organoids, suggesting that ERK activity protects the distal lung epithelia from apoptosis after the loss of centrioles [122]. Whether the levels of ERK activity can counteract p53‐dependent cell death upon the activation of the MSP in other contexts warrants further investigation.

### Centrioles in the developing kidney

The developing embryonic kidney is composed of three types of progenitor cells: mesenchymal, ureteric bud, and stromal progenitors, all of which originate from the mesoderm [157]. Mice lacking Centrosomal protein of 120 kDa (CEP120) and centrosomes specifically in each of these three progenitor populations displayed hypoplastic kidneys at birth (Fig. 5) [123, 124]. At E13.5, the loss of centrosomes decreased the number of progenitor cells by activating the MSP. Furthermore, the loss of centrosomes in the mesenchymal or in the ureteric bud progenitor cells caused premature differentiation into nephron tubular structures and an impaired branching morphogenesis [123]. Of note, the constitutively high ERK activity in these cells did not seem to be sufficient to protect them from cell death and an MSP response [158, 159]. Remarkably, centrosome loss in the both progenitor types resulted in early onset cyst formation and fibrosis in postnatal mice. Interestingly, this phenotype was absent in stromal cells lacking centrosomes [123, 124]. Collectively, these findings suggest that while centrosome loss decreases the pool of progenitor cells via activation of the MSP in all three types of kidney progenitor cells, it promotes growth and cyst formation in those cells that did not undergo apoptosis and differentiated prematurely into tubular epithelial cells.

### Centrioles in the developing skin epithelium

The stratified skin epidermis and ectodermal appendages, such as hair follicles, originate from ectodermal progenitor cells that form a simple epithelial layer at the embryo surface at E9.5. Around E13.5, the epithelial cells undergo a stratification and differentiation program to form a functional skin barrier before birth, and the hair follicles start to form [160, 161]. Deleting *Sas‐4* using a K14‐Cre and the concomitant loss of centrioles from the developing skin epidermis resulted in a thinner epidermis and very sparse and arrested hair follicles due to the activation of the 53BP1‐, USP28‐ and p53‐dependent MSP (Fig. 5), where the additional deletion of *53bp1*, *Usp28* or *p53* rescued the abnormal phenotypes [126]. Intriguingly, only a small fraction of the cells (~ 5%) that lost centrioles around E15.5 died, while the epidermis proceeded through development by adapting its transcriptional program and exhibiting a relatively high tolerance to centriole loss and p53 stabilization [126].

### Centrioles in the developing intestine

The role of centrosomes in the developing intestine has been studied in conjunction with the developing lung in Shh‐expressing endodermal cells depleted of *Sas‐4* [122]. Interestingly, the loss of centrioles in the intestine stabilized p53 but had no apparent effect on the development of the small intestine (Fig. 5), suggesting that centrioles may be dispensable in this context. Furthermore, ERK activity was high in developing intestinal cells and the inhibition of ERK in intestinal organoids dramatically increased apoptosis in acentriolar organoids [122]. Collectively, these studies highlight the different thresholds towards MSP activation after p53 upregulation in tissues derived from the same germ layer, for example in the lung versus intestine, and the potential involvement of protective mechanisms against a severe MSP response.

## Conclusions and perspectives

Growing lines of evidence suggest that while mammalian centrosomes are crucial for providing the centriolar template for the formation of cilia, they are not absolutely essential for cell division *per se*, but rather for a more efficient and timely mitosis. The presence of the dominant centrosome microtubule organizing activity ensures a short mitotic duration and suppresses the activation of a distinct p53‐dependent cell cycle mechanism called the MSP. The MSP may permanently fix differentiated cells that lose centrosome function in a state of non‐proliferation and/or monitor the duration of mitosis in cycling cells and prevent the survival or propagation of cells that experience prolonged mitosis. In this respect, the MSP is similar to how cell cycle checkpoints monitor the order, integrity, and fidelity of key cell cycle events, including cell division. The varying thresholds and responses to the MSP among the different cell types likely lie in how the time spent in mitosis is measured and propagated to downstream signals to elicit cell death or cell cycle arrest. Cancer cells can overcome the consequences of the MSP by mutating p53 or likely other members of the pathway. Notably, the MSP is a potential new cell cycle checkpoint dedicated to measuring mitotic duration, with the typical checkpoint modules of sensors, mediators, and executioners. Future research will focus on establishing this notion and identifying more players within the modules. Finally, it would be interesting to examine the functions of other genes encoding centrosomal proteins, including additional developing tissues and organs, but also extending the studies to adult homeostasis and stem cells, particularly in regenerating tissues.

## Conflict of interest

The authors declare no conflict of interest.

## Author contributions

Writing: CM‐G wrote the review with edits and suggestions from HB; Supervision and Funding Acquisition: HB.
